# Brain stimulation: a therapeutic approach for the treatment of neurological disorders

**DOI:** 10.1111/cns.13769

**Published:** 2021-12-03

**Authors:** Jose Antonio Camacho‐Conde, Maria del Rosario Gonzalez‐Bermudez, Marta Carretero‐Rey, Zafar U. Khan

**Affiliations:** ^1^ Laboratory of Neurobiology CIMES University of Malaga Malaga Spain; ^2^ Department of Medicine Faculty of Medicine University of Malaga Malaga Spain; ^3^ CIBERNED Institute of Health Carlos III Madrid Spain

**Keywords:** deep brain stimulation, invasive brain stimulation, non‐invasive brain stimulation, repetitive transcranial magnetic stimulation, transcranial direct current stimulation, transcranial magnetic stimulation

## Abstract

Brain stimulation has become one of the most acceptable therapeutic approaches in recent years and a powerful tool in the remedy against neurological diseases. Brain stimulation is achieved through the application of electric currents using non‐invasive as well as invasive techniques. Recent technological advancements have evolved into the development of precise devices with capacity to produce well‐controlled and effective brain stimulation. Currently, most used non‐invasive techniques are repetitive transcranial magnetic stimulation (rTMS) and transcranial direct current stimulation (tDCS), whereas the most common invasive technique is deep brain stimulation (DBS). In last decade, application of these brain stimulation techniques has not only exploded but also expanded to wide variety of neurological disorders. Therefore, in the current review, we will provide an overview of the potential of both non‐invasive (rTMS and tDCS) and invasive (DBS) brain stimulation techniques in the treatment of such brain diseases.

## Graphical abstract

Diagrams of representative non‐invasive and invasive brain stimulation techniques. (A) represents the standard figure‐eight transcranial magnetic stimulation (TMS) coil, which is connected to intensity and pulse regulator. (B) exhibits bipolar transcranial direct current stimulation (tDCS) electrodes and current regulator. (C) shows deep brain stimulation (DBS) microelectrodes inserted in brain and connected to a stimulator implanted elsewhere.

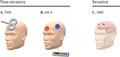

## 1 INTRODUCTION

There are two modalities of brain stimulation: non‐invasive and invasive, and along the time, several techniques have been developed within both categories (Box 1). The non‐invasive stimulation is done by two techniques: transcranial magnetic stimulation (TMS), which was introduced in 1985,^1^ and transcranial electrical stimulation (tES). The transcranial direct current stimulation (tDCS) is the most modern and most used form of tES. These non‐invasive techniques are applied directly through electrodes or magnetic fields on the scalp of the patient to produce electrical currents for the stimulation of brain cells. However, invasive stimulation, such as deep brain stimulation (DBS), involves passing electric current into the subcortical area through surgically implanted electrodes deeper in the brain. Unlike invasive, non‐invasive methods do not require anesthesia and surgical operation, and therefore, these are preferred over invasive methods. Both non‐invasive techniques, rTMS and tDCS, have been used in clinical settings, are already regulated for clinical use in many countries and, currently, are approved by the Food and Drug Administration (FDA). On the other hand, invasive technique, DBS, is also an FDA‐approved treatment and, in the late 1980s, it began to emerge as a life‐changing therapy for patients with involuntary movement disorders.

BOX 1 **Various types of brain stimulation techniques**

**1. Non‐invasive brain stimulation techniques** modulate brain excitability by the application of either magnetic fields over the head or electrical currents directly through electrodes placed on the scalp. There are several modalities of use in both the techniques.

**1.1**. **Transcranial magnetic stimulation (TMS)**

In TMS, short electromagnetic pulses are administered through a magnetic coil. In **repetitive TMS (rTMS),** a figure‐of‐eight coil is used to stimulate precise but relatively superficial locations on the cortex, whereas in **deep TMS (dTMS)** a H‐coil targets broader but deeper brain areas.

**Magnetic seizure therapy (MST)** involves the induction of a seizure by applying high‐intensity magnetic field pulses through a magnetic coil placed on the head. The stimulation is limited to a focused area in the brain, and therefore, it produces minimal effect in surrounding tissues.

**1.2**. **Transcranial electrical stimulation (tES)**

The most modern and used version of tES is **transcranial direct current stimulation (tDCS)**. In tDCS, continuous but low‐intensity current is applied through electrodes (anode and cathode) placed on the scalp. **High‐definition tDCS (HD‐tDCS)** is a variant of this technique and in contrast to tDCS where distribution of electrical current in a target area is relatively diffused; HD‐tDCS devices are used for increased focal stimulation of a target area.

**Cranial electrotherapy stimulation (CES)** is a form of neurostimulation that applies pulsed, low‐intensity current through electrodes placed on anatomical positions around the head, such as earlobes and temples.

**Transcranial random noise stimulation (tRNS)** is achieved by applying an alternating current which varies in frequency and amplitude (within a certain range) throughout the stimulation period. However, **transcranial alternating current stimulation (tACS)** is frequency specific stimulation, and therefore, current is applied at a fixed frequency rather than randomly acquired range of frequencies as in case of tRNS.

**Electroconvulsive therapy (ECT)** involves a brief electrical stimulation of the brain while the patient is under anesthesia. Electrodes are placed at specific sites on the scalp and electrical currents are passed through the brain to produce a brief seizure.

**2. Invasive brain stimulation techniques** generally involve surgery to implant an electrode deep in the brain to deliver electrical pulses at a high frequency. The intensity and frequency of electrical currents are controlled by a generator implanted under the skin of chest.

**Deep brain stimulation (DBS)** involves application of continuous stimulation through a pair of electrodes implanted in a specific area of brain. However, **vagus nerve stimulation (VNS)** implicates the delivery of electrical pulses to the left vagus nerve through a device implanted under the skin.

## 2 NON‐INVASIVE BRAIN STIMULATION

### 2.1 Transcranial magnetic stimulation

TMS is a neuromodulation technique that uses large transient magnetic fields to induce focal electrical fields in a specific brain area, and the availability of sophisticated equipment has made it possible to employ repetitive TMS (rTMS). The effects of rTMS vary depending on the shape of the coil (figure of eight, H coil, double cone coil),^2^ pacing pattern (high frequency, low frequency, theta‐burst), and stimulation site. In fact, TMS is considered as a tool with great therapeutic potential because it is safe and the risk of severe negative side effects upon application is very low.

#### 2.1.1 Mechanism of action

TMS induces short pulses of intracranial electrical current and is applied in several ways: as single pulse, as paired pulse to the same or different brain areas, or as rTMS. Single‐pulse stimulus depolarizes neurons^3^; however, rTMS can induce changes in excitability of the cerebral cortex, locally as well as in neurons at areas far from the stimulation site, along functional anatomical connections.^3^, ^4^ Although underlying mechanisms of the therapeutic outcomes of rTMS application have not been fully elucidated, rTMS can induce changes in cerebral blood flow,^5^ oxygen consumption, cortical activity,^6^ and release of neurotransmitters.^7^, ^8^ Therefore, it has been argued that these functional changes might be associated with positive clinical results.

#### 2.1.2 TMS application to alleviate the symptoms of neurological disorders

For effective rTMS application, adjustments in both spatial and temporal parameters are essential. In literature, for the determination of spatial location of a target in brain, 52% of the studies have used magnetic resonance imaging, 27% scalp measurement, 15% functional magnetic resonance imaging, and 6% hotspot targeting.^9^ Similarly, temporal parameters, which include stimulation frequency, number of pulses per trial, and interval duration between each stimulus, are also diverse. For stimulation frequency, few studies have used low‐frequency stimulation of 1 Hz and most studies have applied a high‐frequency stimulation ranging from 5 Hz (in 14%), 10–19 Hz (in 67%), to more than 20 Hz (in 20%). The stimulus interval time varied from 300 ms to 37,400 ms, and the number of pulses administered in each trial was <10; however, some studies applied more than 20 pulses. Additionally, combining rTMS with concurrent behavioral interventions in some neurological disorders has turned out to be more effective.^10^ Therapeutic benefits of rTMS are summarized in Table 1.

**TABLE 1:** Therapeutic benefits of application of rTMS in neurological disorders

Disorder	Participant size	Stimulation site	Stimulus frequency	Outcome of treatment	Effect size or SMD and *p*‐value	References and comment
Parkinson´s disease	646	M1	High frequency (10–50 Hz)	Long‐term motor function improvement	0.97 (*p* < 0.01)	Yang et al., 2018^14^ (Meta‐analysis of 23 studies)
Alzheimer´s disease	293	DLPFC	Low frequency (1 Hz)	Improvement in memory functions	1.53 (*p* < 0.005)	Chou et al., 2019^25^ (Meta‐analysis of 13 studies)
			High frequency (5–20 Hz)		0.77 (*p* < 0.005)	
	94	DLPFC	High frequency (>1 Hz)	Improvement in cognitive functions	1.00 (*p* = 0.0008)	Liao et al., 2015^26^ (Meta‐analysis of 7 studies)
Attention deficit hyperactivity disorder	43	PFC	High frequency (18 Hz)	Improvement in ADHD symptoms	0.96 (*p* = 0.0009)	Alyagon et al., 2020^33^ (Clinical trial)
	7	PFC	High frequency (10 Hz)	Improvement in ADHD symptoms	0.48 (*p* < 0.05)	Weaver et al., 2012^32^ (Pilot study)
Dyslexia	10	IPL and STG	High frequency (5 Hz)	Improvement in reading performance	0.54 (*p* < 0.001)	Costanzo et al., 2013^35^ (Pilot study)
Autism spectrum disorder	317	DLPFC (16 studies) PFC (3 studies) SMA (3 studies) PMC (1 study) Multiple sites (1 study)	Low to high frequency (0.5–50 Hz)	Significant improvement in repetitive behavior, sociability, and cognitive and executive functions	ND	Khaleghi et al., 2020^42^ (Review of 24 studies)
	339	DLPFC (15 studies) PFC (3 studies) PMC (3 studies) Multiple sites (2 study)	Low to high frequency (>0.5)	Improvement in repetitive and stereotyped behaviors, social behavior, and executive functions	0.29–0.53 (*p* < 0.008)	Barahona‐Correa et al., 2018^41^ (Meta‐analysis of 23 studies)
Chronic pain	682	M1	High frequency (5–20 Hz)	Significant reduction in pain intensity (up to 32%)	ND	Gatzinsky et al., 2020^50^ (Systematic review of 24 studies)
	250	M1	High frequency (5–20 Hz)	Significant pain relief and long‐lasting analgesic effect	ND	Hamid et al., 2019^48^ (Systematic review of 7 clinical trials)
	727	M1	High frequency (5–20 Hz)	Significant pain relief (>30%)	ND	Galhardoni et al., 2015^49^ (Review of 27 clinical trials)

Abbreviations: DLPFC, dorsolateral prefrontal cortex; IPL, inferior parietal lobe; M1, primary motor cortex; ND, not determined; PFC, prefrontal cortex; PMC, premotor cortex; SMA, supplementary motor area; SMD, standardized mean difference; STG, superior temporal gyrus.

##### Parkinson's disease

A progressive degeneration of dopaminergic neurons in the basal ganglia leads to severe impairment in motor functions of patients with Parkinson´s disease (PD). The application of rTMS by several clinical groups found that PD patients improved motor functions upon application of high‐frequency (10 Hz) rTMS in M1 area of motor cortex and most patients showed improvements in bradykinesia.^11^, ^12^, ^13^, ^14^, ^15^ The motor improvements in PD patients were associated with changes in neuronal activity.^16^ Furthermore, a meta‐analysis of 23 studies with total of 646 patients found that the application of rTMS to the motor cortex area of brain produces a significant long‐term improvement in motor functions.^14^

##### Alzheimer's disease

Alzheimer's disease (AD) is a neurodegenerative disease that causes cognitive deficits and is the most common form of dementia. The application of rTMS in AD patients has been shown to improve motor^17^, ^18^ and cognitive functions.^19^, ^20^ The cognitive improvement was observed immediately and one month after the treatment but not after 6 months.^21^, ^22^ Furthermore, the application of high‐frequency (10 Hz) TMS significantly improved cognitive performance in AD patients with mild deterioration,^23^, ^24^ and similarly, meta‐analysis studies found that rTMS is effective in treating cognitive dysfunctions in AD patients.^25^, ^26^

##### Vascular dementia

Vascular dementia is the second most common form of dementia after AD, and it accounts for at least 20% of dementia cases. A study in rats with vascular dementia showed that application of TMS was able to improve spatial learning and memory,^27^ protect pyramidal cells from apoptosis, and promote synaptic plasticity in the CA1 area of the hippocampus.^28^, ^29^ However, the studies in humans are scarce. Nevertheless, a randomized controlled pilot study in 7 patients with vascular disease and mild cognitive deficits without vascular dementia showed that one session of high‐frequency rTMS applied to the left DLPFC improved executive functioning, whereas no effects on any other cognitive functions were observed.^30^ Another study in patients with vascular disease and vascular cognitive impairments but without dementia found that the stimulation of left DLPFC and not left M1 area with 4 sessions of rTMS significantly improved the cognitive ability.^31^

##### Attention deficit hyperactivity disorder

Attention deficit hyperactivity disorder (ADHD) is primarily associated with deficits in attention and executive functions. A pilot study in 9 adolescents and young adults with ADHD found significant improvement after the treatment with high‐frequency (10 Hz) rTMS.^32^ Another pilot study in 43 adult ADHD patients showed that the application of high‐frequency (18 Hz) rTMS for 3 weeks caused significant improvement in ADHD symptoms.^33^ In contrast, a study in adult ADHD patients reported no effect after application of deep TMS (dTMS).^34^ The effect of standard rTMS is more focal and reaches a depth of 0.7 cm, while the effect of dTMS is broader and reaches a significant depth of 3.2 cm. Therefore, it seems that a focal treatment with rTMS is more effective in the treatment of ADHD.

##### Dyslexia

Dyslexia affects at least 5% of school‐aged children and is characterized by difficulty in learning to read and spelling of written texts. Most dyslexics have difficulties in relating alphabet letters to the sounds they symbolize. So far, there is no study with larger number of dyslexia patients. In a study with 10 dyslexics, treatment with high‐frequency (5 Hz) rTMS to areas that are not very active in dyslexics during reading, such as the left superior temporal gyrus and the left inferior parietal lobe, improved both precision and reading speed of the dyslexic adults.^35^

##### Autism spectrum disorder

Autism spectrum disorder (ASD) is a developmental disorder and is characterized by the difficulty in social interaction and emotional recognition, repetitive behaviors, and lack of interest. The prevalence of ASD is estimated at 1 every 110 births with a higher incidence in children.^36^, ^37^, ^38^ In a study, application of low‐frequency (1 Hz) rTMS on DLPFC area of autistic patients caused significant improvements in the process of goal recognition, reduction of motor errors to specific stimuli, and reduction of repetitive and stereotactic behaviors.^39^ Another study showed that autistic youths as well as adults improved their executive functions after the application of high‐frequency (20 Hz) rTMS on the DLPFC.^40^ In the same line, a review of 24 studies with 317 ASD patients and a meta‐analysis of 23 studies with 339 ASD patients found that the application of rTMS improved the ASD symptoms in patients.^41^, ^42^

##### Down syndrome

Down syndrome is a genetic disorder; however, patients with Down syndrome show various neurological symptoms, such as neuromotor abnormalities, reduced learning capacity, cognitive and language alterations, and hampered reading skills.^43^, ^44^, ^45^ The first study with TMS on the motor cortex showed that young people with Down syndrome have normal cortical excitability, but altered cortical synaptic plasticity.^46^ So far, there is no study of TMS application to improve the language and cognitive alterations in Down syndrome.

##### Chronic pain

Chronic pain is a disorder associated with various pathologies and is thought to develop from CNS nerves damage. It has been shown that a single stimulation with high‐frequency TMS produced small (12%) but short‐term reduction in pain intensity, which was not considered as clinically meaningful.^47^ However, a systematic review of 12 randomized clinical trials involving 350 patients with focal or generalized chronic pain found that low‐frequency rTMS stimulation produced no effect, whereas high‐frequency stimulation induced long‐lasting analgesic effect and meaningful relief from chronic pain.^48^ Similarly, other systematic reviews and meta‐analysis have identified that rTMS^49^, ^50^ as well as rTMS combined with exercise^51^ has beneficial effect on relieving patients from chronic pain.

### 2.2 Transcranial direct current stimulation

tDCS is the most used form of electrical stimulation. In comparison with rTMS, tDCS is not as powerful and generates weak stimulus; however, it is relatively easy to use and transport, lot less expensive, and it has low incidence of side effects. The effect of tDCS varies according to the type of current (direct, alternating, pulsed, random noise), polarity (anodal or cathodal), current intensity, and stimulation site.^52^

#### 2.2.1 Mechanism of action

tDCS modulates neural activity by delivering low‐amplitude electrical current through electrodes and therefore causes a change in the cortical excitability. An anodal tDCS stimulation enhances excitatory synaptic transmission by stimulating glutamate transmission and suppressing gamma‐aminobutyric acid (GABA) transmission and that the change in the balance between glutamate and GABA activities leads to modification in functional connectivity between brain regions.^53^, ^54^, ^55^, ^56^ The effect of anodal tDCS stimulation also extends to other brain areas through decrease/increase in axonal release of monoamine transmitters, such as dopamine.^57^ In addition, an anodal tDCS stimulation has been shown to cause induction in long‐term potentiation (LTP),^58^ increase in cAMP accumulation^59^ and mRNA expression,^60^ which are kinds of biological activities that facilitate the processing of cognitive functions.^61^

#### 2.2.2 tDCS application to alleviate the symptoms of neurological disorders

Therapeutic benefits of tDCS are summarized in Table 2.

**TABLE 2:** Therapeutic benefits of application of tDCS in neurological disorders

Disorder	Participant size	Stimulation site	Stimulus current density (mA/cm^2^)	Outcome of treatment	Effect size or SMD and *p*‐value	References and comment
Alzheimer´s disease	146	DLPFC (3 studies) Temporal cortex (3 studies) Temporoparietal areas (1 study)	0.06–0.08 (single session)	Improvement in cognitive performance	0.84 (*p* = 0.002)	Cai et al., 2019^65^ (Meta‐analysis of 7 studies)
	93	DLPFC (3 studies) Temporoparietal areas (1 study) Temporal cortex (1 study)	0.06–0.08	Improvement in cognitive functions	1.35 (*p* < 0.001)	Hsu et al., 2015^67^ (Meta‐analysis of 5 studies)
Parkinson´s disease	325	M1 (9 studies) DLPFC (4 studies) PFC (1 study) Multiple sites (4 studies)	0.028–0.13	Improvement in locomotion	0.36 (*p* = 0.001)	Lee et al., 2019^69^ (Meta‐analysis of 18 studies)
	152	M1 (4 studies) PFC (2 studies) Multiple sites (3 studies)	0.02–0.06	Improvement in gait	0.61 (*p* = 0.005)	Goodwill et al., 2017^70^ (Meta‐analysis of 9 studies)
Attention deficit hyperactivity disorder	241	DLPFC (10 studies) IFG (1 study)	0.02–0.08	Significant improvement in attention, inhibitory control and working memory	ND	Cosmo et al., 2020^88^ (Systematic review of 11 studies)
	169	DLPFC	0.028–0.08	Improvement in inhibitory control and working memory	2.42–2.76 (*p* < 0 0.015)	Salehinejad et al., 2019^91^ (Meta‐analysis of 9 studies
Dyslexia	10	Temporoparietal areas	0.04	Improvement in text accuracy, word recognition speed, perception, and attentional focusing	2.50 (*p* = 0.01)	Lazzaro et al., 2021^84^ (Pilot study)
	63	Temporoparietal areas	0.04	Significant improvement in reading ability	ND	Finisguerra et al., 2019^82^ (Systematic review of 3 studies)
Autism spectrum disorder	84	DLPFC (6 studies) Temporoparietal junction (1 study) Multiple sites (1 study)	0.02–0.08	Significant improvement in repetitive behavior, sociability, and cognitive and executive functions	ND	Khaleghi et al., 2020^42^ (Systematic review of 8 studies)
	69	DLPFC	0.028–0.17	Reduction in ASD symptoms	ND	Osorio et al., 2019^74^ (Systematic review of 5 studies)
	266	DLPFC (10 studies) Temporoparietal junction (3 studies) M1 (2 studies) Multiple sites (4 studies)	0.028–0.08	Improvement in socialization, repetitive behavior, and sensory and cognitive awareness	0.97 (*p* < 0.001)	García‐González et al., 2021^72^ (Meta‐analysis and review of 19 studies)
Epilepsy	328	Temporal lobe (2 studies) Parietal lobe (2 studies) M1 (2 studies) Multiple sites (21 studies)	0.028–0.17	Significantly reduced seizures frequency	ND	Sudbrack‐Oliveira et al., 2019^100^(Systematic review of 27 studies)
	128	Temporal lobe (2 studies) Temporoparietal areas (1) M1 (3 studies) Multiple sites (6 studies)	0.028–0–083	Significantly reduced seizures frequency	ND	Regner et al., 2018^98^(Systematic review of 12 studies)
Cerebral palsy	128	M1	0.028–0.04	Significant improvement in gait, mobility, and balance	ND	Fleming et al., 2018^105^ (Review of 10 studies)
	373	M1	0.028–0.04	Improvement in velocity, stride length, and cadence	3.75–4.48 (*p* < 0.0005)	Saleem et al, 2019^107^ (Review and meta‐analysis of 17 studies)
	178	M1 (8 studies) Cerebellum (1 study)	0.028–0.04	Improvement in gait velocity and step length	0.23 (*p* < 0.01)	Hamilton et al., 2019^106^ (Review and meta‐analysis of 9 studies)
Chronic pain	747	M1	0.025–0.083	Reduction in pain intensity and improvement in quality of life	0.43–0.66 (*p* < 0.05)	O´Connell et al., 2018^47^ (Review and meta‐analysis of 27 studies)

Abbreviations: DLPFC, dorsolateral prefrontal cortex; IFG, inferior frontal gyrus; M1, primary motor cortex; ND, not determined; PFC, prefrontal cortex; SMD, standardized mean difference.

##### Alzheimer's disease

Studies have shown that tDCS can stabilize verbal memory in patients with AD dementia^62^ and enhance the listening comprehension.^63^ The stimulation of left DLPFC with tDCS for 5 days produced significant improvement in immediate and delayed recall performance of a picture memory and that this improvement persisted for one month.^64^ In addition, a meta‐analysis of 7 studies with a total of 146 mild‐to‐moderate AD patients showed that tDCS stimulation significantly improved the cognitive functions.^65^ Similarly, other meta‐analysis studies also found an improvement in cognitive functions of AD patients after tDCS stimulation.^66^, ^67^

##### Parkinson's disease

Several studies have shown that tDCS is beneficial in improving movement disorders in PD patients. A systematic review of 29 studies involving single tDCS session with 256 PD patients and repeated tDCS sessions with 294 PD patients found significant improvement in motor symptoms, including mobility, balance, gait velocity, and falling.^68^ Similarly, meta‐analysis of 18 studies in 325 PD patients and of 9 studies in 152 PD patients revealed that tDCS stimulation significantly improved PD symptoms, including walking performance, gait, and bradykinesia.^69^, ^70^

##### Autism spectrum disorder

An application of tDCS in children and adolescents with ASD has been shown to increase brain functional connectivity^71^ and cause improvement in behavioral and cognitive symptoms.^72^, ^73^, ^74^ Both cathodal and anodal tDCS stimulation are adequate in successfully reducing ASD symptoms even in medication‐resistant patients.^75^, ^76^, ^77^

##### Down syndrome

A study with 22 Down syndrome children of ages between 6 and 12 years showed that the application of 10 sessions of anodal tDCS on the primary motor cortex during the upper limb motor training enhanced motor control for a reach movement.^78^ Similarly, a case report found that anodal tDCS combined with upper limb motor training led to improvement in duration and velocity of movement.^79^ Even though these results are encouraging, there is lack of comprehensive studies on the effects of tDCS application in patients with Down syndrome.

##### Dyslexia

Studies in dyslexic children and adolescents have shown that a treatment with tDCS causes improvement in reading skills and reduction in word reading errors and wordless reading time gap.^80^, ^81^, ^82^, ^83^ A study in 10 dyslexic children further demonstrated that the application of anodal tDCS improved text accuracy, word recognition speed, motion perception, and attentional focusing.^84^ In addition, tDCS stimulation combined with training for reading in children and adolescents with dyslexia produced long‐lasting improvement in reading.^85^ Application of tDCS also improved reading speed and fluency in dyslexic adults.^86^

##### Attention deficit hyperactivity disorder

Several meta‐analysis and other studies in ADHD patients have shown that the tDCS treatment increases brain connectivity and improves behavior, attention, working memory, inhibitory control, and cognitive flexibility.^87^, ^88^, ^89^, ^90^, ^91^ In addition, a study in 37 ADHD patients showed that tDCS causes an improvement in impulsivity symptoms.^92^

##### Epilepsy

Studies in children and adults with focal as well as refractory focal epilepsy have shown that a stimulation with cathodal tDCS decreases epileptiform discharges.^93^, ^94^, ^95^, ^96^ Similarly, several meta‐analysis and systematic reviews found that cathodal tDCS application in epileptic patients with either focal epilepsy or refractory focal epilepsy successfully restrained epileptiform activity and reduced seizure frequency.^97^, ^98^, ^99^, ^100^

##### Cerebral palsy

Cerebral palsy is a permanent movement disorder that is caused by abnormal motor development or damage to the parts of brain that control movement, balance, and posture. Recent studies in children and adolescents with cerebral palsy have shown that tDCS stimulation combined with physiotherapeutic training improves body roll speed, balance, mobility, and walking distance and decreases spasticity and gait.^101^, ^102^, ^103^, ^104^ These studies showed that single tDCS session caused improvement for a short period; however, tDCS treatment sessions ranging from several weeks to few months produced more sustained effect. A treatment with tDCS alone also improved mobility, gait, and balance in pediatric cerebral palsy patients.^105^, ^106^, ^107^

##### Chronic pain

Studies have shown that a treatment with tDCS on the M1 area causes long‐lasting relief in medication‐resistant patients with chronic pain syndrome such as trigeminal neuralgia, post‐stroke pain, back pain, and fibromyalgia.^108^, ^109^ The efficacy of tDCS in alleviating pain has also been shown in patients with multiple sclerosis joint pain,^110^ neuropathic pain,^111^ spinal cord injury,^112^ fibromyalgia,^113^ chronic migraine,^114^ foot pain,^115^ and intra‐abdominal pain.^116^ A meta‐analysis studies further found that a treatment with tDCS reduces chronic pain intensity.^47^

## 3 INVASIVE BRAIN STIMULATION

### 3.1 Deep brain stimulation

DBS treatment implies passing electric current into the subcortical nuclei of the brain through surgically implanted electrodes. In contrast to rTMS and tDCS, DBS treatment in some of the brain nuclei has been shown to produce severe side effects.

#### 3.1.1 Mechanism of action

Although how DBS produces improvements remains not well understood, it has been shown that DBS treatment changes brain activity in a controlled way. The effects of DBS tend to cause excitation in neighboring axons, improvement in microvascular integrity, increase in local cerebral blood flow, and stimulation in astrocytes to release calcium, which can further lead to the release of glutamate and adenosine.^117^ In addition, there is evidence that DBS can induce local and possibly distal proliferation of neurons.^118^ Nevertheless, from a neurophysiological point of view, the "disruption hypothesis" appears to be increasingly accepted. According to this hypothesis, DBS dissociates the input and output signals and causes a disruption in the anomalous flow of information.^119^

#### 3.1.2 DBS application to alleviate the symptoms of neurological disorders

Therapeutic benefits of DBS are summarized in Table 3.

**TABLE 3:** Therapeutic benefits of application of DBS in neurological disorders

Disorder	Participant size	Stimulation site	Stimulus (Hz)	Outcome of treatment	Effect size, SMD, or overall effect and *p*‐value	References and comment
Alzheimer´s disease	132	Fornix (8 studies) NBM (7 studies) VC / VS (1 study)	20–130	Improvement in memory and reduction in cognitive decline	ND	Luo et al., 2021^124^ (Review of 16 studies)
Parkinson´s disease	1189	STN (5 studies) STN/GPI (2 studies) CZI (1 study)	130–167	Improvement in motor function and activities of daily living	2.40–6.36 (*p* < 0.02)	Bratsos et al., 2018^126^ (Meta‐analysis of 8 studies)
	1252	STN (1 study) GPI/STN (9 studies) GPI (2 studies) PPN (2 studies) VIM (2 studies)	25–185	Improvement in motor functions	3.43 (*p* < 0.01)	Mao et al., 2019^127^ (Meta‐analysis of 16 studies)
Essential tremor	1202	VIM	50–200	Improvement in tremor severity (>60%) and quality of life (>56%)	ND	Giordano et al., 2020^131^ (Systematic review of 38 studies)
	430	VIM	50–157	Significant improvement in essential tremor	ND	Flora et al., 2010^130^ (Systematic review of 17 studies)
Epilepsy	328	ANT (20 studies) CMT (7 studies) Hippocampus (10 studies)	60–185	Significant reduction in seizure frequency (>56%)	ND	Zhou et al., 2018^139^ (Review of 37 studies)
	150	ANT (1 study) CMT (2 studies) Hippocampus (4 studies)	10–190	Reduction in seizure frequency	2.26–9.27 (*p* < 0.02)	Sprengers et al., 2017^137^ (Meta‐analysis of 7 clinical trials)
Chronic pain	304	PAG/PVG and/or VPL/VPM	5–162	Significant reduction in pain intensity (upto 60%)	ND	Galafassi et al., 2021^141^ (Systematic review of 11 studies)
	228	PAG/PVG and/or VPL/VPM (18 studies) ACC (2 studies) VS/ALIC (1 study) PLIC (1 study)	5–130	Significant reduction in pain intensity (upto 60%)	ND	Frizon et al., 2020^140^ (Systematic review of 22 studies)
Tourette syndrome	162	GPI (11 studies) Thalamus (4 study) GPI/Thalamus (6 studies)	20–185	Significant reduction in tic severity (>57%)	1.96 (*p* < 0.001)	Coulombe et al., 2018^145^ (Meta‐analysis of 21 studies)
	150	GPI (19 studies) Thalamus (17 studies) GPI/Thalamus (4 studies) ALIC/NAC (7 studies) STN (1 study)	20–185	Significant reduction in tic severity (>52%)	0.96 (*p* = 0.002)	Baldermann et al., 2016^147^ (Meta‐analysis of 48 studies

Abbreviations: ACC, anterior cingulate cortex; ALIC/NAC, anterior limb of the internal capsule/nucleus accumbens; ANT, anterior nucleus of thalamus; CMT, centromedian nucleus of thalamus; CZI, caudal zona incerta; GPI, globus pallidus internus; NBM, nucleus basalis de Meynert; ND, not determined; PAG/PVG, periaqueductal/periventricular gray matter region; PLIC, posterior limb of internal capsule; PPN, pedunculopontine nucleus; SMD, standardized mean difference; STN, subthalamic nucleus; VC/VS, ventral capsule/ventral striatum; VIM, thalamic ventral intermediate nucleus; VPL/VPM, ventral posterior lateral/posterior medial thalamus; VS/ALIC, ventral striatum/anterior limb of the internal capsule.

##### Alzheimer's disease

A case study found that the forniceal DBS in a patient with severe AD symptoms improved the activities of daily living but had no effect on cognition^120^ and a phase II and two‐year follow‐up study in 42 patients with more than 65 years of age and mild AD showed that the application of DBS in the fornix improved memory.^121^, ^122^ Similarly, a review of 16 studies with 174 AD patients and another review of 9 studies with 45 AD patients found that a stimulation with DBS in fornix caused improvement in memory and slowed down the cognitive decline.^123^, ^124^ In addition, application of DBS in entorhinal cortex and nucleus basalis of Meynert has also been shown to be beneficial for improving memory in AD patients.^125^

##### Parkinson's disease

DBS is effectively used in the management of motor functions in PD patients and the most common target areas have been globus pallidus pars interna (GPi) and subthalamic nucleus (STN). Several meta‐analysis studies found that the application of DBS improved motor functions as well as daily living activities.^126^, ^127^, ^128^ In addition, a study of combined effect of DBS in STN and levodopa medication showed that the DBS stimulation and levodopa medication independently improved motor symptoms to a similar extent in PD patients; however, the combined effect was greater than either one of the treatments.^129^

##### Essential tremor

DBS is considered as an effective and safe therapy for essential tremor. Several meta‐analysis studies in essential tremor patients found significant improvement after DBS treatment.^130^, ^131^

##### Autism spectrum disorder

In a case report, application of DBS in basolateral amygdala caused improvement in the core symptoms of ASD and the related self‐injurious behavior in a patient of 13 years of age.^132^ Similarly, in another case report, a 14‐year‐old boy with ASD and self‐injurious behavior treated with DBS in nucleus accumbens showed significant improvement as well.^133^ Nevertheless, there is lack of comprehensive study in a larger number of patients to demonstrate the efficacy of DBS in ASD.

##### Epilepsy

Four patients with partial and generalized epileptic seizures who received DBS treatment in thalamus showed 49% reduction in seizures over a period of 44 months, and one of the patients did not suffer seizures for 15 months.^134^ A multicenter, double‐blind, randomized study in 110 adults with refractory partial seizures showed decrease in seizures for 2 years^135^; however, long‐term follow‐up of the same study further confirmed the efficacy of this therapy even 5 years after the treatment.^136^ In addition, several reviews and meta‐analysis studies have shown that DBS treatment induces significant reduction in seizures frequency in epileptic as well as refractory epileptic patients.^137^, ^138^, ^139^

##### Chronic pain

DBS has been shown to be effective in reducing chronic pain up to 60% in patients.^140^, ^141^ A study in 16 patients with chronic pain showed that DBS‐mediated stimulation of thalamus produced considerable reduction in pain and this effect persisted 36 months after the treatment.^142^ Similar to the treatment in thalamus, a study of DBS in anterior cingulate cortex also found significant improvement in pain, and the effect of the treatment lasted for an average of 18 months^143^ and 39 months after the treatment.^144^

##### Tourette syndrome

Tourette syndrome is a neurodevelopmental disorder characterized by the appearance of involuntary repetitive motor and vocal tics. High percentage of patients also present other brain disorders, such as attention deficit hyperactivity disorder (ADHD) and obsessive‐compulsive disorder (OCD). A meta‐analysis study found that DBS‐mediated stimulation of both the GPi and the thalamic nucleus improved tics and decreased OCD in patients,^145^ and a review of 48 studies in 120 patients with Tourette syndrome found substantial improvement in the severity of tics.^146^ Similarly, other reviews and meta‐analysis studies identified that the stimulation of thalamus, globus pallidus, or nucleus accumbens produced overall improvement in the symptoms of Tourette syndrome.^147^, ^148^, ^149^

## 4 CONCLUDING REMARKS

The success of brain stimulation treatment lies in the availability of an effective tool and the most desirable device would be the one which not only can penetrate deep into the brain and focally modulate a specific region and only that region but also is cheap, portable, and painless, and can be applied in awake, alert humans. However, currently available devices fall short of such expectations. Considering that brain stimulation technologies continue to evolve and advancing rapidly, more versatile tools are expected to develop in near future. Nonetheless, within the currently available non‐invasive devices, tDCS involves passing relatively weak direct current in the brain and is inexpensive and relatively safe. While TMS is more expensive and might occasionally cause a seizure (<1%), it is powerful. In contrast, tDCS cannot cause a seizure and is weak. DBS, which is an invasive technique, is often used as a last resort for treating patients who have shown no relief after other viable therapies, and compared to tDCS and TMS, DBS produces serious side effects. For example, there is high rate of suicide in patients treated with DBS, particularly with stimulation in STN and GPi areas of brain.^150^ Within TMS, tDCS, and DBS techniques of brain stimulation, TMS is the most used in clinical applications. Currently, more than 2000 clinical trials are registered in clinicaltrials.gov for TMS. This number is in fact almost twice of clinical trials registered for either tDCS or DBS. In addition, TMS also supersedes in the number of publications recorded in PubMed. Considering that TMS technology continues to evolve as we have seen with the development of new broad and deep TMS coils, it is likely that TMS may adopt in future and become the most desirable and sophisticated device.

## CONFLICT OF INTEREST

Authors declare no conflict of interest.
